# Oxidative Stress in Migraine—Effect or Cause?

**DOI:** 10.3390/genes17060624

**Published:** 2026-05-29

**Authors:** Oliwia Szymanowicz, Bartosz Słowikowski, Mateusz Konieczny, Dominik Lewandowski, Wojciech Owecki, Marianna Jeżewska, Ulyana Goutor, Paweł P. Jagodziński, Wojciech Kozubski, Jolanta Dorszewska

**Affiliations:** 1 Laboratory of Neurobiology, Department of Neurology, Poznan University of Medical Sciences, 60-355 Poznan, Poland; 76624@student.ump.edu.pl (O.S.); 86063@student.ump.edu.pl (M.K.); 86525@student.ump.edu.pl (D.L.); 86897@student.ump.edu.pl (W.O.); marjez6@st.amu.edu.pl (M.J.); 86648@student.ump.edu.pl (U.G.); 2 Doctoral School, Poznan University of Medical Sciences, 60-812 Poznan, Poland; 3 Department of Biochemistry and Molecular Biology, Poznan University of Medical Sciences, 60-781 Poznan, Poland; bslowikowski@ump.edu.pl (B.S.); pjagodzi@ump.edu.pl (P.P.J.); 4 Student Scientific Society, Poznan University of Medical Sciences, 60-806 Poznan, Poland; 5 Chair and Department of Neurology, Poznan University of Medical Sciences, 60-355 Poznan, Poland; wkozubski@ump.edu.pl

**Keywords:** oxidative stress, molecular markers, environmental factors, pathophysiology, migraine

## Abstract

Migraine is a complex neurovascular disorder with a multifactorial pathophysiology involving genetic, metabolic, and environmental factors. Increasing evidence indicates that oxidative stress plays a key role in the development of migraine; however, it is unclear whether oxidative imbalance acts primarily as a causal factor or occurs as a consequence of migraine-related processes. Oxidative stress, defined as an imbalance between reactive oxygen species production and antioxidant defense mechanisms, contributes to neuronal hyperexcitability, mitochondrial dysfunction, and neuroinflammation—key mechanisms underlying migraine pathogenesis. Studies have shown elevated markers of oxidative damage and altered antioxidant enzyme activity in migraine patients. Simultaneously, metabolic and inflammatory changes associated with migraine may further exacerbate oxidative imbalance, suggesting a bidirectional relationship. Furthermore, genetic factors such as *SOD2*, *GPX1*, and *CAT* significantly influence susceptibility to oxidative stress and migraine. The *CALCA* gene, encoding CGRP, links oxidative stress mechanisms with neurogenic inflammation and activation of the trigeminovascular system. This article reviews the current evidence regarding the role of oxidative stress in migraine and discusses its relationship to molecular and genetic mechanisms. Particular attention is given to genes involved in oxidative pathways, mitochondrial function, and inflammatory responses, which may help explain individual susceptibility and variability in clinical presentation.

## 1. Introduction

Migraine is one of the most common chronic neurological disorders, significantly impacting patients’ quality of life and their social and professional functioning [1,2]. It is estimated to affect over one billion people worldwide each year and occurs more frequently in women than in men [3,4]. Migraine is characterized by recurrent headache attacks with specific clinical features, most commonly unilateral, throbbing, of moderate to severe intensity, and aggravated by routine physical activity. These attacks are often accompanied by autonomic symptoms such as nausea, vomiting, and hypersensitivity to external stimuli, including light, sound, and odors. Some patients also experience a migraine aura, which consists of transient neurological symptoms, most often visual, but also sensory or motor [5,6]. Migraine, therefore, represents a significant social and public health problem with substantial epidemiological impact [7]. Attacks frequently lead to temporary disability, reduced work productivity, and limitations in social and family life. Consequently, migraine generates considerable economic costs, both direct (related to treatment) and indirect (resulting from absenteeism and decreased productivity) [1,8].

Despite extensive research, the pathogenesis of migraine remains complex and not fully understood. The current consensus is that migraine results from the interaction of multiple factors, including genetic predisposition as well as immunological, hormonal, biochemical, and environmental mechanisms.

## 2. Theories of Migraine

### 2.1. The Vascular Theory

One of the earliest theories explaining migraine development was the vascular theory, which proposed that migraine results from alternating changes in the diameter of cerebral blood vessels. According to this hypothesis, the initial phase of migraine, particularly the aura, is caused by vasoconstriction and transient ischemia, whereas the headache phase results from subsequent vasodilation and activation of pain receptors within the vessel walls [9]. However, contemporary research has demonstrated that the vascular theory does not fully explain all aspects of migraine. It has been observed that changes in cerebral blood flow and vessel diameter do not consistently correlate with pain intensity, and in some patients, they may occur independently of clinical symptoms. Currently, vascular mechanisms are considered to play a secondary role in a more complex process involving primarily neuronal and neurochemical disturbances [10].

### 2.2. The Neurogenic Theory

The neurogenic theory currently represents the predominant explanation of migraine pathophysiology, with activation of the trigeminovascular system playing a central role [11]. This system comprises trigeminal nerve fibers that innervate the meningeal blood vessels. Activation of these structures leads to the release of numerous neuropeptides, including calcitonin gene-related peptide (CGRP), substance P, and neurokinin A. These mediators induce vasodilation, increase vascular permeability, and contribute to the development of so-called sterile neurogenic inflammation. As a result, pain receptors are activated, and nociceptive signals are transmitted to higher centers of the nervous system [12,13].

Another important aspect of the neurogenic theory is the involvement of brainstem structures responsible for pain modulation and autonomic regulation. Dysfunction within these regions may lead to a lowered pain threshold and increased sensitivity to external stimuli. Furthermore, enhanced cortical excitability has been observed in individuals with migraine, which facilitates the initiation of migraine attacks [14].

### 2.3. The Theory of Cortical Spreading Depression

The theory of cortical spreading depression (CSD) is a key element in the current understanding of migraine pathophysiology, particularly in migraine with aura [15]. This phenomenon refers to a wave of massive depolarization of neurons and glial cells slowly spreading through the cerebral cortex, followed by a prolonged phase of neuronal inhibition. CSD begins as a result of a sudden ionic imbalance within neurons, leading to their rapid depolarization. This leads to an intense influx of sodium and calcium ions into the cells and an efflux of potassium ions into the extracellular space. This phenomenon is accompanied by an excessive release of excitatory neurotransmitters, particularly glutamate, which further enhances the depolarization of neighboring neurons and facilitates wave propagation. The CSD wave spreads through the cerebral cortex at a speed of approximately 2–6 mm per minute, which correlates with the clinically observed gradual progression of aura symptoms, such as visual disturbances moving across the patient’s field of vision. The depolarization phase is followed by a period of inhibition of neuronal activity, which can last from several minutes to even several dozen minutes and is responsible for transient neurological symptoms [16,17,18]. The CSD phenomenon is accompanied by significant changes in cerebral blood flow. Initially, a short-term increase in blood flow (hyperemia) is observed, followed by a longer phase of decreased flow (oligemia). Importantly, these changes do not reach levels that lead to permanent brain tissue damage, but they can affect neuronal function and promote the development of clinical symptoms. Cortical spreading depression also plays a significant role in the initiation of a migraine attack. CSD results in the release of numerous mediators, such as potassium ions, protons, nitric oxide, and reactive oxygen species, which can diffuse into the meninges and activate the trigeminal nerve endings. This leads to stimulation of the trigeminovascular system and the initiation of the cascade of inflammatory and pain processes characteristic of migraine [19,20].

### 2.4. Neurotransmitters in Migraine

Another important aspect involves the role of neurotransmitters, particularly serotonin, which plays a key role in regulating vascular tone and modulating the transmission of nociceptive stimuli. Migraine is associated with dysfunction of the serotonergic system, including decreased serotonin levels, which may lead to increased neuronal excitability and facilitate the activation of pain pathways. Other neurotransmitters, such as dopamine, glutamate, and gamma-aminobutyric acid (GABA), also contribute by influencing the balance between excitation and inhibition within the central nervous system [21,22].

### 2.5. Genetic Factors of Migraine

Genetic factors also play a significant role in migraine pathophysiology. Migraine frequently aggregates in families, suggesting a hereditary component, and in certain rare forms, specific mutations in genes encoding ion channel proteins have been identified. The best characterized example is hemiplegic migraine, in which mutations in genes such as *CACNA1A*, *ATP1A2*, and *SCN1A* have been reported. Dysfunction of these ion channels leads to abnormal ion transport across neuronal membranes, resulting in increased neuronal excitability and facilitating the development of CSD. In more common forms of migraine, inheritance is polygenic, involving multiple genes that influence neuronal excitability, vascular function, and neurotransmitter metabolism [23,24].

In summary, migraine is a disease with a complex etiology, in which the interaction of multiple pathophysiological mechanisms plays a key role. Current models suggest that a migraine attack results from disturbances in the functioning of the central nervous system, leading to activation of the trigeminovascular system, inflammatory processes, and biochemical and metabolic changes. No single theory fully explains all aspects of this disease; therefore, an approach that integrates various mechanisms into a single, coherent pathophysiological model is most justified.

## 3. Genetic and Epigenetic Factors of Oxidative Stress in Migraine

Oxidative stress has emerged as a significant but still not fully defined factor of migraine pathophysiology. Although numerous studies report elevated levels of reactive oxygen species (ROS) and lipid peroxidation products in migraine patients, the interpretation of these findings remains inconsistent, particularly in the context of causality. Current evidence suggests that oxidative imbalance is not an isolated phenomenon but rather an outcome of broader metabolic and neuronal dysregulation, including mitochondrial dysfunction and altered neurovascular coupling [25,26].

**Figure 1:**
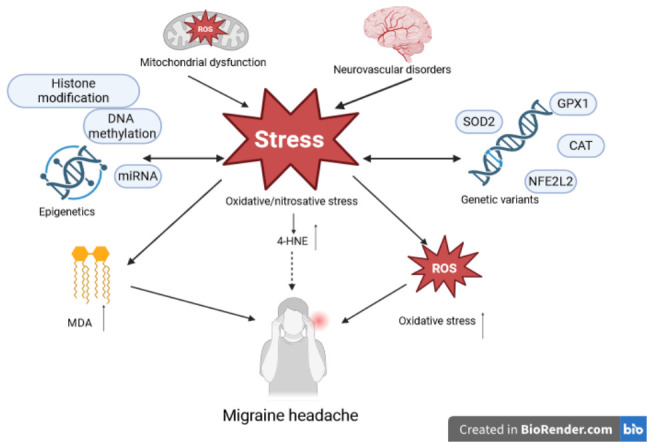
Potential involvement of oxidative/nitrosative stress in migraine attacks. MDA, malondialdehyde; 4-HNE, 4-hydroxynonenal; ROS, reactive oxygen species; SOD2, superoxide dismutase; GPx1, glutathione peroxidase; CAT, catalase; NFE2LE, NFE2-like bZIP transcription factor 2. (↑) elevated level.

From a genetic perspective, susceptibility to oxidative stress in migraine appears to be polygenic and subtle rather than driven by single high-impact variants (Figure 1). Several single-nucleotide variants (SNVs) in genes encoding key antioxidant enzymes have been investigated, including *SOD2* (rs4880), *GPX1* (rs1050450), *CAT* (rs1001179), and *NFE2L2* (rs6721961) [27]. These enzymes play central roles in redox homeostasis: SOD2 catalyzes the dismutation of superoxide radicals into hydrogen peroxide within mitochondria, GPX1 reduces hydrogen peroxide and lipid peroxides using glutathione (GSH), while CAT further detoxifies hydrogen peroxide into water and oxygen. Functional variants in these genes may therefore alter the efficiency of ROS neutralization at multiple levels of the antioxidant defense system [28,29]. For instance, the *SOD2* (rs4880) polymorphism affects mitochondrial targeting efficiency and enzymatic activity, with the Ala variant generally associated with altered ROS handling [30]. Similarly, the *GPX1* Pro198Leu substitution (rs1050450) has been linked to reduced enzyme activity under oxidative conditions, potentially impairing the detoxification of peroxides [31]. In the case of *CAT*, promoter variants such as −262C>T (rs1001179) may influence transcriptional activity and, thus, overall antioxidant capacity [32]. Beyond these enzymes, polymorphisms in regulatory genes such as *NFE2L2* (encoding NRF2), a master transcriptional regulator of antioxidant responses, have also been implicated. Variants affecting NRF2 signaling may lead to a blunted induction of cytoprotective genes under oxidative stress conditions [33]. From a translational perspective, NRF2 signaling may represent a particularly attractive therapeutic target in migraine. Unlike approaches focused on single antioxidant enzymes, NRF2 functions as a master regulator of cellular antioxidant responses and controls the coordinated expression of multiple cytoprotective genes, including *SOD2*, *GPX1*, and *CAT.* Therefore, modulation of NRF2 activity may potentially restore global redox homeostasis rather than correcting isolated oxidative abnormalities. Although NRF2-targeted interventions in migraine remain largely experimental, increasing evidence from neurological and neurodegenerative disorders suggests that pharmacological activation of NRF2 may reduce oxidative damage, mitochondrial dysfunction, and neuroinflammatory responses [27,33]. The mentioned SNVs have also been associated with migraine risk; however, their individual effects are modest and often population-dependent [27]. Despite these mechanistic links, the clinical relevance of individual SNVs remains limited. Reported associations between these variants and migraine are often modest and inconsistent across populations, reflecting differences in genetic backgrounds, environmental exposures, and study designs [34]. This lack of reproducibility suggests that these variants do not act as primary drivers of disease but rather contribute to a cumulative genetic context that modulates redox resilience.

Polygenic background gains functional relevance at the level of mitochondrial metabolism, which represents a common ground between genetic susceptibility and oxidative stress. Mitochondria are the primary source of intracellular ROS under physiological conditions, mainly through electron leakage at complexes I and III of the electron transport chain [35]. Even subtle impairments in oxidative phosphorylation may therefore shift the balance toward increased superoxide production, particularly in energetically demanding cells such as neurons. In the context of migraine, defective mitochondrial function has been associated with reduced ATP availability, impaired ion homeostasis, and increased oxidative burden. These alterations are particularly relevant for neuronal excitability, as ATP-dependent ion pumps, including Na^+^/K^+^-ATPase, are essential for maintaining membrane potential. Energy deficits may thus lower the threshold for depolarization and facilitate the propagation of CSD, a key electrophysiological event underlying migraine aura. At the same time, excessive ROS production can directly modulate ion channels and signaling pathways, further enhancing neuronal hyperexcitability and promoting neurogenic inflammation [36,37,38].

Despite these mechanistic insights, several key aspects of oxidative stress in migraine remain unresolved. In particular, the variability of clinical presentation and the inconsistent reproducibility of genetic and biochemical findings suggest that static models based solely on inherited variants or metabolic defects may be insufficient [27,34]. Moreover, oxidative stress in migraine is often transient and context-dependent, fluctuating between ictal and interictal periods, which further complicates its interpretation as either a cause or a consequence of the disease [39]. This limitation points to the possible influence of regulatory mechanisms capable of integrating genetic predisposition with environmental and metabolic inputs. In this context, epigenetic processes emerge as a possible link, suggesting an interesting involvement of gene regulation in modulating cellular responses to oxidative stress over time. Rather than acting independently, epigenetic alterations may determine how efficiently cells respond to oxidative changes, thereby affecting both vulnerability to and recovery from migraine attacks [40].

Epigenetic mechanisms, including DNA methylation, histone modifications, and non-coding RNAs, regulate gene expression without altering the DNA sequence and are highly sensitive to environmental and metabolic impacts [41]. Importantly, oxidative stress itself can influence these mechanisms, for instance, by modifying DNA methyltransferase activity or altering chromatin structure, thereby linking redox imbalance with gene regulation [40,42].

DNA methylation represents one of the most extensively studied epigenetic modifications in migraine. For example, hypomethylation of the *CALCA* promoter at specific CpG sites has been reported in migraine patients, potentially contributing to increased CGRP expression and enhanced neurogenic inflammation. Altered methylation patterns have also been reported in genes involved in inflammatory signaling (*STAT6*, *SERPING1*), vascular regulation (*LRP1*, *NOS3*), and neuronal excitability (*TRPM8*, *CACNA1A*, *MEF2D*) [40,43,44,45]. More detailed insights come from epigenome-wide association studies (EWAS), which have identified differentially methylated regions (DMRs) associated with migraine. For example, a genome-wide methylation study by Gerring et al. [45] identified 62 DMRs in whole blood, enriched near genes involved in solute transport and hemostasis, suggesting a link between epigenetic regulation and vascular and metabolic pathways. Notably, no single CpG site reached genome-wide significance, reinforcing the notion that methylation changes in migraine are subtle and distributed rather than driven by strong locus-specific effects. More recent integrative analyses combining methylation quantitative trait loci data with GWAS have provided stronger evidence for specific genes. A large-scale study identified 169 CpG sites mapping to 68 genes with potential causal relevance to migraine, including the mentioned above *LRP1* (participates in vascular integrity and neuronal signaling), *CALCA* (encodes CGRP, a key mediator of neurogenic inflammation and migraine pain), *TRPM8* (sensory transduction and pain perception), and *MEF2D*. These findings suggest that DNA methylation may modulate canonical migraine pathways rather than introduce entirely novel mechanisms. However, even in this context, the directionality of effects remains complex, with both hyper- and hypomethylation at different CpG sites associated with either increased or decreased disease risk [44]. Additional support for the functional relevance of methylation changes comes from longitudinal clinical studies. In patients with chronic migraine undergoing medication withdrawal, dynamic alterations in DNA methylation were associated with a reduction in headache frequency, indicating that epigenetic states may shift in parallel with clinical improvement rather than remain fixed over time. This observation strongly supports the concept of epigenetic plasticity in migraine and argues against interpreting methylation patterns as stable diagnostic biomarkers [46]. Finally, indirect evidence linking methylation to migraine also emerges from studies of one-carbon metabolism. Variability in genes such as *MTHFR*, which regulate folate-dependent methylation processes, has been associated with migraine susceptibility and treatment response, suggesting that systemic methylation capacity may influence disease expression [47].

In addition to DNA methylation, post-transcriptional regulation mediated by microRNAs (miRNAs) represents another epigenetic mechanism that potentially links oxidative stress with migraine pathophysiology. As is broadly known, miRNAs are short non-coding RNAs that regulate gene expression by targeting messenger RNA, leading to translational repression or degradation. Due to their rapid responsiveness to environmental and metabolic cues, miRNAs are particularly well suited to mediate transient changes in cellular homeostasis, including those induced by oxidative stress. Several miRNAs have been implicated in migraine, although the available evidence remains heterogeneous and cohort-dependent [48]. Among the most consistently reported is miR-155, which has been shown to be upregulated in patients with migraine, particularly in chronic forms, and is strongly associated with inflammatory signaling pathways. Functional studies further indicate that miR-155 may promote neuroinflammation and central sensitization, for example, through modulation of SIRT1 and downstream inflammatory cascades [49]. Other miRNAs discussed in the context of migraine include miR-34a and members of the let-7 family, which are implicated in the regulation of apoptosis, mitochondrial function, and neuronal differentiation. These processes are closely linked to oxidative stress, suggesting that miRNA dysregulation may contribute to the cellular response to redox imbalance rather than acting as an isolated pathogenic factor [50,51]. Importantly, epigenetic mechanisms may also have translational relevance because, unlike inherited genetic variants, they are potentially reversible and responsive to environmental or pharmacological interventions. This raises the possibility that future therapeutic approaches targeting DNA methylation patterns or miRNA-mediated regulation could modulate oxidative stress responses, neuroinflammation, and neuronal excitability in a more dynamic and personalized manner [40,50].

Taken together, current evidence indicates that oxidative stress in migraine should not be viewed as an isolated pathogenic trigger but rather as an emergent property of complex interactions between genetic predisposition and epigenetic regulation. While variants in antioxidant-related genes and mitochondrial dysfunction provide a mechanistic basis for altered redox homeostasis, their individual contributions appear modest and insufficient to explain the full clinical heterogeneity of the disease. Epigenetic mechanisms, including DNA methylation and miRNA-mediated regulation, offer a more dynamic framework, linking environmental and metabolic factors with gene expression changes. However, the available data suggest that these modifications are subtle and often reversible, rather than constituting stable disease-specific markers. This is further supported by the lack of reproducible biomarkers and the variability observed across cohorts and disease stages. Importantly, oxidative stress itself may act as both a driver and a consequence of these regulatory processes, forming a bidirectional network in which mitochondrial dysfunction, redox imbalance, and gene regulation reinforce each other. Within this framework, migraine can be conceptualized as a disorder of impaired adaptive capacity, where the inability to effectively respond to metabolic and oxidative challenges leads to neuronal hyperexcitability and attack susceptibility. Considering the central role of mitochondrial dysfunction in ROS generation and neuronal energy imbalance, mitochondria-oriented therapeutic strategies may also represent a promising future direction in migraine management.

## 4. Markers of Oxidative Damage in Migraine

### 4.1. Malondialdehyde

**Figure 2:**
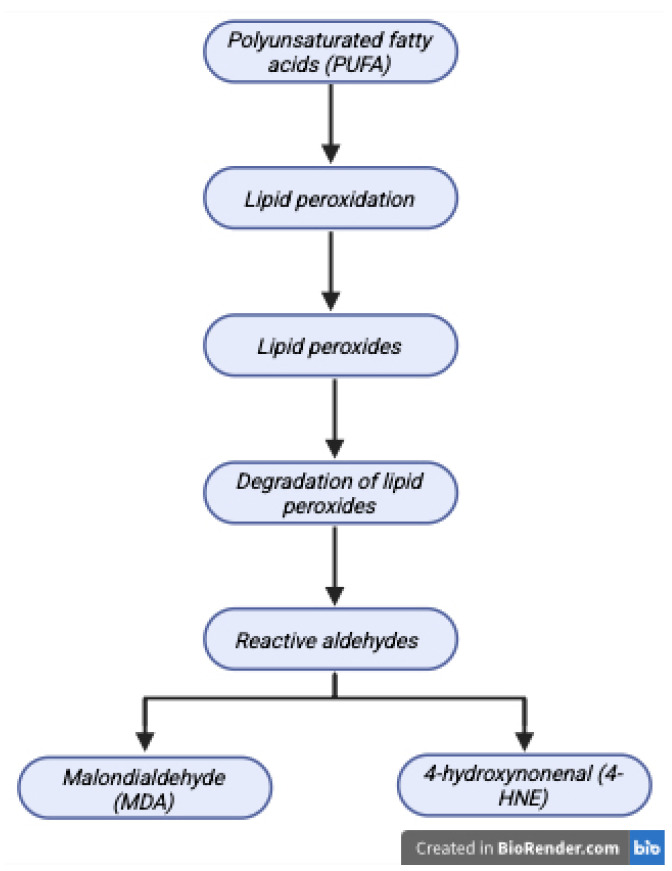
Peroxidation of polyunsaturated fatty acids to malondialdehyde and 4-hydroxynonenal.

Malondialdehyde (MDA) is one of the best-known products resulting from the peroxidation of polyunsaturated fatty acids (Figure 1 and Figure 2). This compound, a reactive aldehyde, exhibits significant biological properties—it not only reflects the degree of oxidative damage to lipids, but can also interact with macromolecules such as DNA and proteins, contributing to changes with potential mutagenic and atherogenic significance [52].

Clinical studies have shown that patients with migraine may have elevated levels of MDA and nitric oxide (NO) metabolites compared to healthy individuals. This phenomenon is explained by the increased production of reactive ROS, which promotes both oxidative and nitrosative stress [53]. Based on this, it can be assumed that migraine patients experience an oxidative–antioxidant imbalance. This is manifested by increased levels of markers of oxidative damage, such as MDA and NO, with a simultaneous decrease in the body’s antioxidant capacity, both in terms of non-enzymatic mechanisms and the activity of antioxidant enzymes. It has also been noted that the severity of these changes may correlate with the frequency of headaches—a higher number of headache days per month is associated with an increase in the level of oxidative stress biomarkers and a decrease in total antioxidant capacity. Comparative studies indicate that MDA concentrations are higher in patients with chronic migraine than in those with episodic migraine, accompanied by lower total non-enzymatic antioxidant capacity. Other analyses have shown that migraineurs have higher MDA levels than patients with tension-type headaches, while no significant differences are observed between those with tension-type headaches and the healthy population. Furthermore, higher MDA values were noted in patients with migraine with aura compared to those with migraine without aura, which may suggest a greater contribution of oxidative stress in this form of the disease [54]. These observations indicate that MDA may be a significant supportive biomarker in migraine diagnosis, especially in clinically ambiguous cases. It has also been noted that increased levels of oxidative stress in migraineurs may persist even between attacks, suggesting a chronic nature [54,55].

### 4.2. TBARS

The TBARS test is commonly used to assess lipid peroxidation in biological fluids and serves as an indicator of oxidative stress. The method is based on the reaction of lipid peroxidation products, primarily MDA, with thiobarbituric acid (TBA), which leads to the formation of a colored complex measured spectrophotometrically. The measurement is performed under acidic conditions and at high temperature, which allows for the release of MDA from a stable precursor used as a standard. The test is simple and rapid, and it demonstrates reproducible results across biological samples [56]. Studies indicate that migraine patients have higher levels of TBARS in blood and plasma compared to healthy individuals [57]. Similarly, elevated levels of TBARS and NOx were found in the urine of migraineurs before treatment. Following flunarizine therapy, TBARS concentrations decreased, although they remained higher than in healthy controls. These results suggest that flunarizine limits oxidative reactions in patients with migraine [58].

### 4.3. 4-Hydroxynonenal

4-Hydroxynonenal (4-HNE) may exacerbate cellular damage induced by oxidative stress. The toxicity of this compound is primarily related to its ability to form protein bonds, which leads to the inactivation of certain antioxidant enzymes and further increases oxidative stress, and consequently, the initiation of apoptosis. The presence of 4-HNE is also associated with the development of aging-related diseases and neurodegenerative disorders [59].

4-HNE can form adducts with DNA and proteins, which disrupt the functioning of numerous signaling pathways, including mechanisms controlling apoptosis. 4-HNE-protein complexes are a significant indicator of lipid peroxidation, and their increasing concentration in tissues and cerebrospinal fluid with age contributes to the development of neurodegenerative diseases, such as Alzheimer’s and Parkinson’s disease, as well as ophthalmological disorders, hearing loss, and cancer.

Analysis of 4-HNE concentrations is difficult due to its high reactivity and instability [60]. At the same time, this compound plays a role in modulating the cellular response to oxidative stress, which may influence the neuroinflammatory mechanisms associated with migraine [59].

Studies have shown that HNE levels are significantly elevated in migraine patients compared to healthy individuals [60,61].

### 4.4. Lipid Peroxides

Lipid peroxides (PerOx) and alpha lipoic acid (ALA) may also be important markers of oxidative stress in migraine. A study of 32 patients with frequent migraine episodes found significant changes in oxidative stress markers. Nearly all patients had decreased levels of ALA and lactate, while almost half had elevated levels of PerOx, indicating increased oxidative stress. Furthermore, many patients had decreased total antioxidant capacity (TAC) and thiol concentrations. These results support the role of oxidative stress and metabolic disturbances in migraine and suggest that ALA may be a potential biomarker of the disease [62].

### 4.5. Markers of Nitrosative Stress

**Figure 3:**
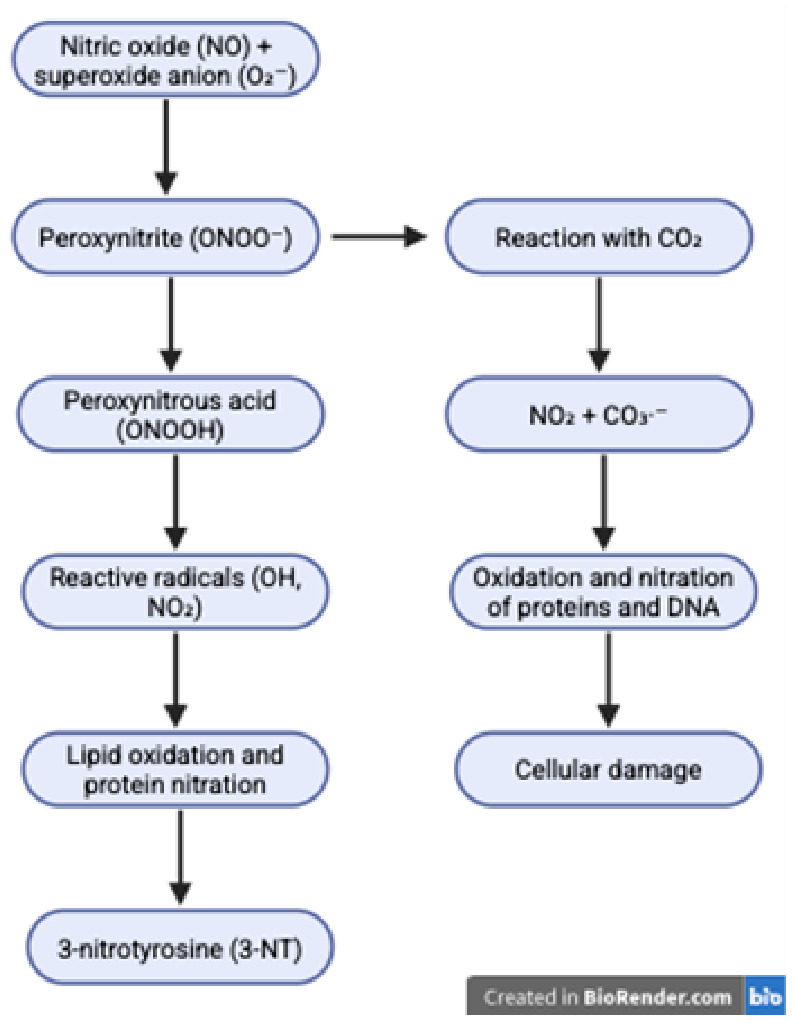
Formation of toxic nitrogen compounds.

The reaction of NO with superoxide (O_2−_) produces peroxynitrite (ONOO^−^), a toxic metabolite that has the potential to disrupt a wide range of biological molecules (Figure 3). Peroxynitrite can be converted to peroxynitrous acid (ONOOH), and both molecules have the potential to cause direct molecular damage. ONOOH is further degraded into reactive radicals, including the single-electron hydroxyl (OH ·) and nitrogen dioxide (NO_2_), thereby contributing to oxidative stress through lipid oxidation and protein nitration. This is evidenced by the production of 3-nitrotyrosine (3-NT), a “tracer” of nitrosative stress in the brain parenchyma. Furthermore, ONOO^−^ can interact with carbon dioxide (CO_2_) to form NO_2_ and carbonate radicals (CO_3_^−^), which then oxidize and nitrate proteins and DNA, thereby exacerbating cellular damage. Furthermore, elevated NO levels have been shown to impair key cellular processes, including glycolysis and oxidative phosphorylation, by inducing protein S-nitrosation, which can lead to mitochondrial dysfunction [63].

Excessive production of NO, particularly through inducible nitric oxide synthase (iNOS) in immune cells, leads to the formation of reactive nitrogen species (RNS), which may exhibit neurotoxic effects and contribute to the development of diseases such as stroke and multiple sclerosis. iNOS is induced by proinflammatory cytokines in cells such as macrophages and microglia, and the produced NO serves a dual role—it participates in the elimination of pathogens, but its excessive levels promote demyelination and axonal damage.

NO reacts with superoxide anion (O_2_^−^) to form peroxynitrite (ONOO^−^), which is then converted into reactive species capable of lipid oxidation and protein nitration. The product of these processes is 3-nitrotyrosine (3-NT), a marker of nitrosative stress. Additionally, reactive nitrogen derivatives exacerbate cellular damage and disrupt metabolic processes, including glycolysis and oxidative phosphorylation, which can lead to mitochondrial dysfunction [63].

Nitric oxide is an important signaling molecule involved in the pathogenesis of migraine. NO donors, such as nitroglycerin, can trigger migraine attacks, and increased activity of the NO pathway is observed in migraine patients. NO is synthesized by three synthase isoforms (nNOS, eNOS, and iNOS), and its dysregulation is considered a significant component of migraine pathogenesis. Therefore, NOS inhibitors represent a potential therapeutic target [64].

However, a meta-analysis of nineteen studies involving over 1000 patients found no significant differences in NO concentrations in plasma, serum, or urine. Nevertheless, higher levels were observed during migraine attacks, in patients with aura, and under the influence of dietary factors [57,65].

## 5. Antioxidants, Oxidative Stress, and Migraine

The exact molecular mechanism of migraine is still difficult to assess; however, recent research focused on antioxidants provides valuable insight into the role of oxidative stress in migraine’s pathogenesis. When the organism experiences oxidative stress, a large number of oxidants, such as ROS or RNS, are produced in mitochondria. Under physiological conditions, it is catalyzed by antioxidant factors, and intracellular ROS are kept at a safe low level [66]. The antioxidative scavenging system consists of enzymatic agents, including superoxide dismutase (SOD), catalase (CAT), and glutathione peroxidase (GPx), as well as non-enzymatic agents, such as GSH, and several dietary factors, such as vitamins A, C, and E [67]. Genetic variations in the genes encoding SOD, CAT, and GPx can affect gene expression, leading to reduced ROS detoxification and possibly increased migraine risk [3].

SOD is an endogenous antioxidant scavenging oxygen free radicals. SOD2 isoforms are the only enzymes expressed in mitochondria capable of catalyzing the dismutation of superoxide into oxygen and hydrogen peroxide. In this way, they limit superoxide accumulation from oxidative metabolism [68]. It is the only enzymatic biomarker whose activity seems to be consistently reduced in migraine patients, including during the interictal measurements [69]. Findings indicating lower activity of SOD and higher levels of oxidative stress indicators were confirmed in a meta-analysis of 19 studies assessing the role of oxidative/nitrosative pathways in patients with migraine compared to a healthy control group [57,70]. In the context of SOD activity, it is important to separate migraine with aura (MA) and migraine without aura (MO) as two diseases with diverse pathophysiology. Significantly higher activity of the described enzyme is observed in MA patients [71]. The possible explanation is that *SOD2* genetic variants can cause defective control of the oxidative phenomena linked to cortical spreading depression—strictly associated with aura—leading to the overstimulation of trigeminal neurons [72]. The described difference in SOD activity can also be related to ROS-induced vasoconstriction and the subsequent increase in oxidative stress, observed especially in patients suffering from MA [71].

The majority of the ROS antioxidation in the cytoplasm is carried out through GPx, and the isoform GPx1 is a crucial endogenous, selenium-dependent antioxidant [66]. It is one of the main enzymes catalyzing the reduction of GSH in the glutathione redox cycle. This enzyme serves multiple roles, including protecting the biofilm from ROS-mediated damage and maintaining normal cellular function. As GPx1 activity is strictly connected to the level of GSH—GSH provides reducing equivalents for hydrogen peroxide removal—it has an important role in protecting neurons from oxidative neurotoxicity. It is noteworthy that the age-related decline in GSH correlates with impaired cognitive function in aging processes [73].

CAT is a ubiquitous antioxidant enzyme controlling the intracellular hydrogen peroxide concentration by catalyzing the breakdown of hydrogen peroxide into water and oxygen [68]. Research conducted by Aytac et al. [74] aimed to assess clinical differences among migraine patients based on their level of oxidative stress. Patients with high levels—defined by low CAT activity and high plasma MDA concentration, the final product of lipid peroxidation and a widely used marker of oxidative stress—were characterized by an increased risk of white matter hyperintensities (WHM). It is widely known that migraine patients are at risk of developing WHM, which are characterized by axonal loss, glial hypocellularity, enlarged extracellular space, and increased extracellular water fraction. The study proved that the CAT serum level significantly decreased in migraine patients with WHM compared to healthy controls and migraine patients without WHM. These findings suggest that, despite a potentially multifactorial pathophysiology of WHM, oxidative stress plays a crucial role in generating such lesions.

Several studies aim to evaluate the importance of oxidative stress in pathophysiology, but their methods are very often divergent, and thus, the results are contradictory. The research performed by Yigit et al. [75] focused on measuring antioxidant levels in migraine patients. Results showed that they had significantly higher concentrations of total oxidant status (TOS), oxidative stress index (OSI), and MDA. Another important finding is the fact that total antioxidant status (TAS) and CAT activity were lower than in the control group. Even though numerous studies examining the role of oxidative stress in migraine have been inconsistent, almost all of them show that at least one marker is abnormal. Another piece of evidence supporting the involvement of oxidative stress in migraine pathogenesis is trigger factors, including: stress (71.5%), alcohol (38.5%), sleep changes (33%), weather changes (26.2%), dehydration (25.3%), and physical activity (19%) [27,76]. The majority of the listed factors can possibly generate oxidative stress. A study performed by Togha et al. [53] investigated serum biomarkers of oxidative stress in patients with episodic and chronic migraine. It is noteworthy that patients suffering from chronic migraine had significantly lower non-enzymatic antioxidant capacity and higher oxidative stress than patients with episodic migraine and healthy controls. This finding correlates with the phenomenon that as the frequency of headache days increased, MDA levels rose, and the levels of SOD and CAT decreased. Described changes in laboratory values might possibly predict the progression from episodic to the chronic stage of migraine and support the hypothesis of oxidative stress’s important role in the disease’s pathophysiology. The aforementioned enzymatic and non-enzymatic factors are crucial, but not the only biomarkers of oxidative stress; levels of heavy metals can possibly be increased in migraine patients. The most important one is free iron—being a heavily pro-oxidant factor—which was found to accumulate in the periaqueductal gray matter in patients with episodic and chronic migraine; more research is needed to assess its exact role [77,78]. Evaluating specific markers of oxidative stress in migraine is difficult, as studies are very often inconsistent—probably due to different methodologies, patient inclusion criteria, and certain biomarkers’ dependence on the migraine cycle—some of them are elevated only during an attack, but not interictally [69].

Studies assessing the impact of dietary nutrients in migraine patients can also serve as a source of information concerning oxidative stress’s role in migraine, as many of these nutrients are well known for their antioxidant properties. One of the best examples is vitamin E—the main antioxidant of the lipid phase of the cell—whose levels were proven to be lower in migraine patients than in the healthy control group [79]. Vitamin C is widely known as a strong antioxidant that effectively slows the oxidation of diverse substrates, such as lipids, carbohydrates, and nucleic acids. Its additional effect enhances the regeneration of other antioxidants, such as vitamin E [80]. Unfortunately, there are no randomized clinical trials assessing prophylactic treatment with vitamin C in migraine patients. Research performed by Visser et al. [81] examined 35 participants and showed that prophylactic supplementation with N-acetylcysteine, vitamin E, and vitamin C combined, daily for three months, resulted in lower frequency, severity, and duration of headaches. Another study’s results are consistent with those mentioned before—an uncontrolled open-label clinical study examined the impact of an antioxidant formula, which included vitamin C and Pinus radiata bark extract. Daily administration of this formula reduced the frequency and severity of headaches after 3 months, and the patients taking this formula for 12 months experienced more than 50% reduction in frequency and severity of headaches [82]. These studies only indicate the possible impact of antioxidants, and thus, the role of oxidative stress in migraine pathogenesis; however, the exact role of dietary antioxidants should be examined in randomized clinical trials.

## 6. Calcitonin Gene-Related Peptide as a Diagnostic and Therapeutic Factor in Migraine

CGRP is one of the key neuropeptides that has been extensively studied and is thought to play an important role in the pathophysiology of migraine. It is a multifunctional neuropeptide broadly distributed throughout both the central and peripheral nervous systems (CNS and PNS). CGRP exists in two main isoforms, α-CGRP and β-CGRP, which are encoded by the *CALCA* and peripheral nervous system genes, respectively [83,84]. The α-CGRP isoform predominates in the CNS and PNS, whereas β-CGRP is more prominently expressed in the enteric nervous system [84]. CGRP binds primarily to a heteromeric receptor complex composed of the calcitonin receptor-like receptor (CLR) and receptor activity-modifying protein 1 (RAMP1). CGRP expression in the CNS is widespread and includes the spinal cord, brainstem, cerebral cortex, cranial nerve nuclei, amygdala, and locus coeruleus. Particularly relevant in the context of migraine is its presence in the trigeminal ganglia (TG) and dorsal root ganglia (DRG). Regions with especially high expression of CGRP receptors include the cerebellum, nucleus accumbens, substantia nigra, and spinal cord. High-affinity CGRP receptors are also present in various peripheral tissues, such as the liver, spleen, and immune cells [83].

At the cellular level, CGRP exerts its effects through activation of G protein-coupled receptors (GPCRs), leading to an increase in intracellular cAMP levels and activation of protein kinase A (PKA). This results in modulation of ion channel activity, including phosphorylation and activation of potassium channels, which leads to hyperpolarization of smooth muscle cells and reduced Ca^2+^ influx, ultimately causing vasodilation [85]. In addition, CGRP can modulate glutamatergic transmission, contributing to changes in gene expression and neuroplasticity associated with central sensitization [83].

The trigeminovascular system is considered the primary peripheral site of CGRP action in migraine. Following activation, trigeminal afferent fibers release CGRP within the dura mater and pia mater, where it interacts with blood vessels, immune cells, glial cells, and nerve fibers. This leads to vasodilation and neurogenic inflammation. Pain signals originating from meningeal vessels are then transmitted via the trigeminocervical complex to the thalamus and subsequently to higher cortical regions, where pain is perceived [83]. It is worth noting that reactive oxygen species (ROS) have been suggested to stimulate CGRP production and release within the trigeminal system, providing a potential link between oxidative stress and migraine mechanisms [86].

The role of CGRP in CSD is supported by studies showing that blockade of CGRP or its receptor not only reduces migraine frequency but also decreases susceptibility of the cortex to CSD. Intravenous infusion of CGRP has also been shown to induce migraine-like headache attacks in patients, supporting its potential causal role in migraine pathophysiology. Clinical evidence also indicates that CGRP levels are often elevated in patients with migraine. Increased concentrations have been observed in venous blood draining from the head, as well as in saliva, tears, plasma, and cerebrospinal fluid, both during and between migraine attacks [83,86]. However, these findings are not entirely consistent, as some studies have failed to demonstrate significant changes in CGRP levels [83,84]. Moreover, measurements in body fluids do not directly reflect concentrations at sites of action, and the short half-life of CGRP complicates reliable detection. Consequently, the utility of CGRP as a biomarker for migraine remains limited [83].

One of the strongest pieces of evidence supporting the role of CGRP in migraine pathophysiology is the clinical efficacy of therapies targeting this neuropeptide. Currently, several CGRP-targeted treatments have been approved for both acute and preventive management of migraine [83].

Among the most important therapies are monoclonal antibodies directed against CGRP or its receptor. Four agents (eptinezumab, fremanezumab, galcanezumab, and erenumab) have been approved for the prevention of migraine. Three of these (fremanezumab, eptinezumab, and galcanezumab) bind directly to CGRP, whereas erenumab acts by blocking the CGRP receptor. Their mechanism of action involves reducing CGRP activity, which results in decreased frequency of migraine attacks. However, treatment response is heterogeneous; some patients experience substantial improvement, while others derive limited therapeutic benefit [83,87].

Gepants are small-molecule antagonists targeting the CGRP receptor [83]. They act by blocking the CGRP receptor [87]. Currently, ubrogepant and rimegepant are indicated for acute migraine therapy, while atogepant and rimegepant are used in preventive treatment [83]. In addition, an intranasal formulation, zavegepant, has been approved for acute treatment [88]. Importantly, both monoclonal antibodies and gepants are thought to modulate rather than completely inhibit CGRP signaling [83].

Classical antimigraine drugs, such as triptans, also exert indirect effects on the CGRP pathway. By acting as agonists at serotonin receptors (5-HT_1_B/D/F), they inhibit CGRP release from trigeminal nerve endings, which correlates with relief of migraine pain.

Despite the strong evidence supporting the effectiveness of CGRP-targeted therapies, the precise sites of action of both CGRP and these drugs remain unclear. It is generally believed that CGRP exerts its effects through both the CNS and the PNS. Further research is needed to deepen the understanding of these mechanisms and improve future treatment approaches.

## 7. Can Oxidative Stress Cause a Migraine Attack?

Even though there are several theories of migraine, the pathogenesis of this neurological disorder remains incompleatly understood. Traditional hypotheses oscillate between vascular and neuronal views. However, more recent research suggests a combined “vessel-to-neuron phenomenon” constituting a complex interplay of various factors [12,89,90,91]. Moreover, accumulating evidence indicates that oxidative stress plays an important role in migraine pathophysiology (Table 1) [57,92,93]. Reports show that migraine triggers involving intense sensory stimuli, such as loud sounds, blue light, odors, and perfumes containing phthalates, contribute to the increase in oxidative stress [69,89,93]. Multiple mechanisms triggering oxidative stress have been described, including neuroinflammation and activation of microglia, activation of neuronal nicotinamide adenine dinucleotide phosphate (NADPH) oxidase, calcium overload and excitotoxicity, and mitochondrial disturbances—high rates of energy production, toxicity, and altered mitochondrial membrane properties [94].

As we mentioned earlier, the neurovascular theory of migraine involves the trigeminal system [95]. Indeed, mitochondrial oxidative stress of the trigeminal ganglia, along with excessive Ca^2+^ influx, is an important factor contributing to migraine etiology. A study by Yazğan et al. [96] shows that one potential pathway includes the transient receptor potential melastatin 2 (TRPM2), a major pain transducer associated with Ca^2+^ influx. TRPM2-mediated pain and neurotoxicity contribute to intensified oxidative stress and inflammation, irreversible mitochondrial dysfunction, and Ca^2+^ dysregulation. On the other hand, an imbalance in extracellular and intracellular ion concentrations influences the propagation of CSD, leading to impaired synaptic activity and dysregulation of the trigeminovascular system [17,97]. For instance, intracellular Ca^2+^ overload triggers transient oxidative stress, promoting injurious signaling via transient receptor potential subtype anchor protein 1 channels [70,98]. Concomitantly, CSD facilitates the opening of the neuronal pannexin1 (Panx1) megachannel followed by activation of caspase-1, and, indirectly, nuclear factor κB [99]. Nuclear factor κB induces a significant inflammatory reaction, leading to the release of cytokines, including tumor necrosis factor (TNF)-α and interleukins, which can induce trigeminal nerve stimulation and migraine symptoms [100,101]. Importantly, the interaction between oxidative stress and inflammation appears to be bidirectional. Reactive oxygen species not only induce direct cellular damage but also act as signaling molecules capable of activating redox-sensitive inflammatory pathways, particularly nuclear factor κB (NF-κB). Activation of NF-κB promotes transcription of proinflammatory mediators, including TNF-α, IL-1β, and IL-6, which further enhance oxidative stress through stimulation of mitochondrial ROS production and activation of NADPH oxidase. This positive feedback loop may contribute to sustained trigeminovascular activation and central sensitization observed in migraine. In parallel, mitochondrial dysfunction associated with impaired oxidative phosphorylation may amplify inflammatory signaling by increasing ATP depletion, calcium dysregulation, and release of damage-associated molecular patterns. These mechanisms collectively support the concept that oxidative stress and neuroinflammation act synergistically rather than independently in migraine pathophysiology. Another view of oxidative stress in migraine underscores that elevated synthesis of reactive oxygen species, nitric oxide metabolites, and proinflammatory mediators may lead to peroxidation of cell membrane phospholipids and damage to intracellular molecules. This effect causes alterations in normal cellular functioning and is implicated in migraine etiology via loss of receptor function, neurogenic inflammation, disruption of signaling pathways, hyperexcitability of cortical neurons, trigeminovascular system activation, and CSD (Table 1) [71,101,102]. Concomitantly, CSD might also be associated with altered mitochondrial function, generating oxidative stress as a causative factor of migraine [70,98,103]. Malkov et al. [104] postulate that oxidative stress triggers metabolic collapse, including abnormal oxidation of NADPH, silencing of synaptic transmission, and massive neuronal depolarization, thereby strengthening the view that reactive oxygen species accumulation initiates CSD. In contrast, Shatillo et al. [105] suggest that induction of CSD is followed by downstream propagation of oxidative stress within the trigeminal nociceptive system, combined with activation of the trigeminovascular system. Moreover, the authors show that oxidative stress may enhance CGRP release in neurons of the dorsal root ganglion. Indeed, another research confirms that reactive oxygen species influence expression of the CGRP gene in trigeminal glia following CSD and neurogenic inflammation [106]. Similarly, evidence indicates that the relationship between cerebral vasospasm and oxidative stress in migraine is not fully elucidated. Administration of an antioxidant agent reduced oxidative stress without preventing nitric oxide-mediated vasodilatation, suggesting that oxidative stress is likely to occur after alterations in vessel diameter or via a different mechanism [58,107].

On the other hand, some researchers postulate a more interrelated view of the cause-and-effect phenomenon of oxidative stress in migraine. Namely, CSD enhances reactive oxygen species production, which, in turn, induces neuronal excitability, leading to higher CSD susceptibility and a reduced CSD threshold. Concomitantly, CSD increases TNF-α levels, which contribute to elevated neuronal excitability [108,109,110]. Interestingly, in preclinical models, insulin-like growth factor-1 (IGF-1) significantly protects against CSD by mitigating oxidative stress and microglial neuroinflammation [108,109,110,111]. Moreover, recent evidence underscores that the Transient TRPA1 ion channel may be an important factor in migraine pathogenesis, with a dual role in oxidative stress. It seems that the TRPA1 channel might be activated by oxidative stress, but it may also participate in generating, sensing, and transducing oxidative stress. Interestingly, reports indicate that TRPA1 activators include well-known migraine triggers. Furthermore, studies show that activated TRPA1 ion channels induce the release of CGRP via an autocrine mechanism, causing neurogenic inflammation [94,112,113,114,115]. Indeed, the reactive oxygen species/TRPA1/CGRP axis appears to be a potential therapeutic target in migraine, as administration of either a TRPA1 antagonist or an antioxidant inhibited CSD in animal models [86,116,117].

In conclusion, it seems that oxidative stress is both a cause and consequence of migraine.

**Table 1:** Role of oxidative stress in the pathogenesis of migraine.

Migraine Pathophysiology	Triggering Factors	Molecular Parameter	Migraine Attack	References
Oxidative stress	Loud sounds, blue light, odors, and perfumes containing phthalates	-	↑	[93]
Oxidative stress	Neuroinflammation and activation of microglia Excitotoxicity, or Mitochondrial disturbances	Neuronal nicotinamide adenine dinucleotide phosphate (NADPH) oxidase	↑	[94]
Neurovascular theory involves the trigeminal system	Mitochondrial oxidative stress of the trigeminal ganglia	Excessive Ca^2+^ influx Transient receptor potential 2-mediated (TRPM2)	↑	[95,96]
Cortical spreading depression (CSD)	Impaired synaptic activity and dysregulation of the trigeminovascular system Trigeminal nerve stimulation	Neuronal Pannexin1 (Panx1) Cytokines pro-inflammatory, including tumor necrosis factor (TNF)-α and interleukins	↑	[70,97,98,99,100]
Oxidative stress and trigeminovascular system activation, and CSD—feedback	Elevated synthesis of reactive oxygen species	Cell membrane phospholipids Calcitonin gene-related peptide (CGRP)	↑	[101,102,103,104,105,106]
Oxidative stress—dual role in oxidative stress	Participate in generating, sensing, and transducing oxidative stress	Transient receptor potential ankyrin 1 (TRPA1) ion channel Reactive oxygen spe-cies/TRPA1/CGRP axis	↑	[94,112,113,114,115]

(↑)—triggers a migraine attack and/or intensifies migraine pain.

## 8. Conclusions

Migraine is a multifactorial disease with an incompletely understood pathophysiology. No single theory fully explains all aspects of the disease, so research into its pathophysiology is ongoing. It is currently believed that oxidative stress may be involved in the onset of migraine headaches. However, the results of studies on oxidative stress in migraine are inconsistent.

Moreover, it seems that oxidative stress in migraine should not be viewed as an isolated pathogenic factor but rather as an emergent component of complex interactions among genetic and environmental predispositions and epigenetic regulation.

Future studies should therefore focus on integrative, multi-layered approaches combining genomics, epigenomics, and metabolic profiling, ideally in longitudinal designs, to better capture the dynamic nature of these interactions and identify clinically relevant targets for personalized therapeutic strategies.

It should also be emphasized that oxidative stress exhibits interrelated interactions with other factors in migraine etiology, creating a complex loop of stimulation leading to the induction of migraine symptoms. Particularly important appears to be the reciprocal interaction between oxidative stress and neuroinflammatory signaling, where ROS-mediated activation of inflammatory pathways and cytokine release may further exacerbate mitochondrial dysfunction, neuronal hyperexcitability, and trigeminovascular sensitization.

However, future therapeutic approaches may increasingly focus on upstream regulators of oxidative balance and neuronal adaptation, particularly NRF2-mediated antioxidant signaling, epigenetic modulation, and mitochondrial protection. Such strategies may better address the complex and dynamic nature of migraine pathophysiology and support the development of personalized therapeutic interventions.

## Data Availability

No new data were created or analyzed in this study.

## Abbreviations

The following abbreviations are used in this manuscript:

CGRP	Calcitonin gene-related peptide
CSD	Cortical spreading depression
GABA	Gamma-aminobutyric acid
ROS	Reactive oxygen species
SNVs	Single nucleotide variants
EWAS	Epigenome-wide association studies
DMRs	Differentially methylated regions
MDA	Malondialdehyde
NO	Nitric oxide
TBA	Thiobarbituric acid
4-HNE	4-Hydroxynonenal
PerOx	Lipid peroxides
ALA	Alpha lipoic acid
TAC	Total antioxidant capacity
NO_2_	Nitrogen dioxide
CO_2_	Carbon dioxide
iNOS	Nitric oxide synthase
RNS	Reactive nitrogen species
3-NT	3-Nitrotyrosine
SOD	Superoxide dismutase
CAT	Catalase
GPx	Glutathione peroxidase
MA	Migraine with aura
MO	Migraine without aura
GSH	Glutathione
WHM	White matter hyperintensities
TOS	Total oxidant status
OSI	Oxidative stress index
TAS	Total antioxidant status
CNS	Central nervous systems
PNS	Peripheral nervous systems
CLR	Calcitonin receptor-like receptor
RAMP1	Receptor activity-modifying protein 1
TG	Trigeminal ganglion
DRG	Dorsal root ganglia
GPCRs	G protein-coupled receptors
PKA	Protein kinase A
NADPH	Neuronal nicotinamide adenine dinucleotide phosphate
TRPM2	Transient receptor potential melastatin 2
TNF-α	Tumor necrosis factor-α
IGF-1	Insulin-like growth factor-1
